# Effects of body condition score and estrus phase on blood metabolites and steroid hormones in Saanen goats in the tropics

**DOI:** 10.14202/vetworld.2020.833-839

**Published:** 2020-05-04

**Authors:** Pradita Iustitia Sitaresmi, Budi Prasetyo Widyobroto, Sigit Bintara, Diah Tri Widayati

**Affiliations:** 1 Department of Animal Breeding and Reproduction, Faculty of Animal Science, Universitas Gadjah Mada, Yogyakarta, Indonesia; 2 Department of Animal Production, Faculty of Animal Science, Universitas Gadjah Mada, Yogyakarta, Indonesia

**Keywords:** blood serum, body condition score, estrus phase, hormones, Saanen goat

## Abstract

**Background and Aim:**

Body condition scores (BCSs) assessment can be a potential management tool for the improvement of animal performance. The study investigated whether blood metabolic (protein, glucose, blood urea nitrogen, and cholesterol) and steroid hormonal (progesterone, estrogen, and cortisol) parameters are affected by the BCS and estrous status in Saanen goat.

**Materials and Methods:**

Blood samples were collected from three groups of mature, dry, and non-pregnant female goats with different BCSs: 2-2.9 (BCS 2), 3-3.9 (BCS 3), and 4-4.9 (BCS 4) on a BCS scale ranged from 1 to 5. Blood serum was collected (n = 160) to determine the blood metabolic profile and steroid hormone levels, depending on the follicular status.

**Results:**

The results demonstrate a variation in the serum metabolite and hormone (glucose, BUN, protein, estrogen, and cortisol) levels at different BCSs and at different phases of the estrous cycle. The hormonal profiles were significantly different (p<0.05) between the estrous cycle phases. The results suggest that BCSs were associated with blood metabolic profiles and steroid hormone levels.

**Conclusion:**

As it can be concluded, an association between steroid hormone levels and the phases of the estrous cycle existed in Saanen goats.

## Introduction

Saanen is one of the dairy goat breeds recently introduced in Indonesia. Goat productivity is influenced by extrinsic factors such as nutritional status and feed availability [1,2]. The negative effects of energy imbalance can be reduced by controlling the body condition score (BCS) [3]. The BCS also affects hypothalamic activity and certain reproductive hormones [4]. Function of hypothalamic–pituitary–gonadal axis (HPG) is affected by energy imbalance, which could alter the secretion of some reproduction steroid hormones. Reproductive function is associated with alterations in energy and endocrine system [5]. Blood parameters, along with the BCS, are good indicators of the nutritional and health status of animals and could serve as reliable predictors that help in preventing a decline in productivity and health status of the animals and are reliable predictors that could contribute in reproductive function [6].

In Indonesia, low goat productivity can be attributed to thermal stress and feed scarcity, along with some tropical climate conditions. Optimizing of productivity in dairy goats requires precise information about the nutritional status that can be provided by BCS [7]. In tropical countries, crossbreeding programs for temperate breeds of dairy goats, such as the Saanen, are established to improve their adaptability and maintain productivity. The present study was performed under field conditions, and its objective was to determine and describe the effects of the BCS and estrus phase on blood metabolites and endocrine traits.

The study is novel since the relationships between different BCSs and blood metabolic and hormonal profiles in each phase of the estrous cycle in Saanen goat have never been studied in the tropical climate. Moreover, the study was conducted without estrus synchronization. Studies related to the control of the reproductive cycle of dairy goat have become important in recent times. Moreover, a regulation has been implemented that prohibits the use of hormones for this purpose in future, Arguello [8] stated that control of the goat reproductive cycle is important research due to the use of hormones will be not allowed in the future especially in Europe.

The present study aims at highlighting how BCS and estrus phase affect the concentration of certain blood metabolites and the profiles of steroid hormones in Saanen goats.

## Materials and Methods

### Ethical approval

The procedures applied throughout this study have been approved by the Animal Care and Use Committee of Faculty of Veterinary Medicine, Universitas Gadjah Mada, No. 002/EC.FKH/Ket/2019.

### Data collection

The study was conducted from August to November 2018 at the center for livestock breeding and forage of animal feed, BPPTU HTP, located in Baturaden, Purwokerto, Indonesia (7°NL, 109°WL). The experiments were conducted using mature Saanen female goats (3-4 years old) (n=160). Goats were categorized according to their BCS into the following groups: Thin for BCS ranging from 2 to 2.9 (BCS 2) (n=48), normal for BCS ranging from 3 to 3.9 (BCS 3) (n=40), and fat for BCS > 4 (BCS 4) (n=72). Only healthy animals were included in the study. The goats had free access to water and mineral salts and received a complete mixed diet in group pens. All the groups received the same diet. The BCSs ranged from 1 to 5, where 1 indicated very thin and 5 indicated obese [9].

Blood samples were collected every morning at 6 am from August to October 2018. The goats were bled through the jugular vein, and 10 ml of blood was collected in a tube containing EDTA. Serum was separated and stored at -20°C [6]. Blood metabolic profiles (protein, glucose, BUN, and cholesterol) were studied using a UV spectrophotometer (Microlab 300, Italy). The hormonal profiles (progesterone, estrogen, and cortisol) were determined using an ELISA kit (DRG Instruments GmbH, Germany). The kit included horseradish peroxide, which was used as a conjugate for binding to the coated antibody [5]. Estrous cycles were determined using vaginal smears and ultrasonography and by assessing vaginal acidity [10], based on which the animals were divided into two groups – luteal and follicular phases. Data were collected from the luteal phase group (n=125) on days 10-11 and from the follicular phase group (n=35) on days 19-21.

### Statistical analysis

Data were analyzed using a 3×2 factorial, in which three groups of BCS and two phases in estrouscycle were used to assess the effects of BCS and estrus phase on blood metabolite and steroid hormone levels of Saanen goat. All data are presented as mean ± SD.

## Results

The results from this study show significant differences (p<0.05) in blood serum protein, glucose, blood urea nitrogen (BUN), estrogen, and cortisol among different groups. Protein concentration in BCS 2, 3, and 4 was 1.05±0.33, 1.18±0.06, and 1.25±0.09 mmol/L, respectively, as shown in Table-1. Glucose concentration in BCS 2, 3, and 4 was 9.76±1.65, 13.29±1.91, and 11.69±2.39 mmol/L, respectively, as shown in Table-2. BUN concentration in BCS 2, 3, and 4 was 6.33±1.41, 5.87±1.54, and 5.56±1.49 mmol/L, respectively, as shown in Table-3. Estrogen concentration in BCS 2, 3, and 4 was 243.52±49.64, 277.52±71.55, and 2.66±69.87 pmol/L, respectively, as shown in Table-4. Cortisol concentration in BCS 2, 3, and 4 was 104.97±29.51, 63.82±18.48, and 68.53±26.04 nmol/L, respectively, as shown in Table-5. The results from this study also shown that not significantly different (p>0.05) in blood serum cholesterol and progesterone among different groups. Cholesterol concentration in BCS 2, 3, and 4 was 11.20±2.04, 11.51±1.92, and 11.06±2.11 mmol/L, respectively, as shown in Table-6. Progesterone concentration in BCS 2, 3, and 4 was 26.99±15.14, 24.46±15.14, and 25.76±1.53 mmol/L, respectively, as shown in Table-7. The BCS 2 group, which represented thin animals with poor nutrition, showed the lowest serum protein levels and the highest BUN and cortisol levels, as shown in Tables-1, 3, and 5. The BCS 3 group, which represented a normal BCS condition with balanced nutrient utilization, showed the highest glucose and estrogen levels, as shown in Tables-2 and 4.

**Table-1:** Protein levels of Saanen goats in each body condition score during the phases of estrous cycle (mean±SD).

BCS	Phase	n	Protein (mmol/L)	p BCS	p Phase
BCS 2 (2-2.99)	Follicular	10	1.01±0.33	0.00**	
	Luteal	38	1.05±0.14		
	Total	48	1.05±0.09^a^		
BCS 3 (3-3.99)	Follicular	10	1.16±0.08		
	Luteal	38	1.18±0.06		
	Total	48	1.18±0.06^b^		
BCS 4 (4-5.00)	Follicular	10	1.26±0.06		
	Luteal	38	1.25±0.09		
	Total	48	1.25±0.09^c^		
Phase	Follicular	35	6.96±1.23		0.455
	Luteal	125	7.04±0.81		
	Total	160	7.02±0.92		

a,b,c Total means with different superscripts within a column differs significantly (p<0.05). BCS=Body condition score

**Table-2:** Glucose levels of Saanen goats in each body condition score during the phases of estrous cycle (mean±SD).

BCS	Phase	n	Glucose (mmol/L)	p BCS	p Phase
BCS 2 (2-2.99)	Follicular	10	8.55±1.12	0.00**	
	Luteal	38	10.08±1.62		
	Total	48	9.76±1.65^a^		
BCS 3 (3-3.99)	Follicular	10	13.27±1.96		
	Luteal	38	13.56±1.92		
	Total	48	13.29±1.91^b^		
BCS 4 (4-5.00)	Follicular	10	12.69±1.85		
	Luteal	38	11.43±2.36		
	Total	48	11.69±2.39^c^		
Phase	Follicular	35	11.67±2.78		0.638
	Luteal	125	11.53±2.41		
	Total	160	11.57±2.48		

a,b,c Total means with different superscripts within a column differs significantly (p<0.05). BCS=Body condition score

**Table-3:** Blood urea nitrogen levels of Saanen goats in each body condition score during the phases of estrous cycle (mean±SD).

BCS	Phase	n	BUN (mmol/L)	p BCS	p Phase
BCS 2 (2-2.99)	Follicular	10	6.34±1.41	0.06*	
	Luteal	38	6.33±1.42		
	Total	48	6.33±1.41^a^		
BCS 3 (3-3.99)	Follicular	10	6.59±1.69		
	Luteal	38	5.63±1.44		
	Total	48	5.87±1.54^b^		
BCS 4 (4-5.00)	Follicular	10	5.45±1.57		
	Luteal	38	5.59±1.48		
	Total	48	5.56±1.49^c^		
Phase	Follicular	35	6.03±1.60		0.93
	Luteal	125	5.82±1.48		
	Total	160	5.87±1.51		

a,b,c Total means with different superscripts within a column differs significantly (p<0.05). BCS=Body condition score, BUN=Blood urea nitrogen

**Table-4:** Estrogen (E2) levels of Saanen goats in each body condition score during the phases of estrous cycle (mean±SD).

BCS	Phase	n	E_2_ (pmol/L)	p BCS	p Phase
BCS 2 (2-2.99)	Follicular	10	296.46±67.74	0.02**	
	Luteal	38	229.61±32.56		
	Total	48	243.52±49.64^a^		
BCS 3 (3-3.99)	Follicular	10	355.72±68.98		
	Luteal	38	251.45±50.96		
	Total	48	277.52±71.55^b^		
BCS 4 (4-5.00)	Follicular	10	356.45±69.53		
	Luteal	38	242.60±47.25		
	Total	48	266.31±69.87^c^		
Phase	Follicular	35	339.12±72.22**		0.00**
	Luteal	125	241.13±44.72**		
	Total	160	262.28±65.86		

a,b,c Total means with different superscripts within a column differs significantly (p<0.05),

** total means with different superscripts within a row differs significantly (p<0.01). BCS=Body condition score

**Table-5:** Cortisol levels of Saanen goats in each body condition score during the phases of estrous cycle (mean±SD).

BCS	Phase	n	Cortisol (nmol/L)	p BCS	p Phase
BCS 2 (2-2.99)	Follicular	10	92.98±9.57		
	Luteal	38	111.49±29.54		
	Total	48	104.97±29.51^a^		
BCS 3 (3-3.99)	Follicular	10	49.89±10.05		
	Luteal	38	69.52±17.14		
	Total	48	63.82±18.48^b^	0.00**	
BCS 4 (4-5.00)	Follicular	10	68.40±16.18		
	Luteal	38	68.56±28.21		
	Total	48	68.53±26.04^c^		
Phase	Follicular	35	70.95±17.68**		0.03**
	Luteal	125	80.99±32.79**		
	Total	160	78.07±30.94		

a,b,c Total means with different superscripts within a column differs significantly (p<0.05),

** total means with different superscripts within a row differs significantly (p<0.01). BCS=Body condition score

**Table-6:** Cholesterol levels of Saanen goats in each body condition score during the phases of estrous cycle (mean±SD).

BCS	Phase	n	Cholesterol (mmol/L)	p BCS	p Phase
BCS 2 (2-2.99)	Follicular	10	11.49±1.81	0.424	
	Luteal	38	11.13±2.11		
	Total	48	11.20±2.04		
BCS 3 (3-3.99)	Follicular	10	11.87±1.90		
	Luteal	38	11.39±1.95		
	Total	48	11.51±1.92		
BCS 4 (4-5.00)	Follicular	10	10.90±1.81		
	Luteal	38	11.10±2.19		
	Total	48	11.06±2.11		
Phase	Follicular	35	11.35±1.83		0.593
	Luteal	125	11.18±2.10		
	Total	160	11.22±2.04		

^a,b,c^ Total means with different superscripts within a column differs significantly (p<0.05). BCS=Body condition score

**Table-7:** Progesterone levels of Saanen goats in each body condition score during the phases of estrous cycle (mean±SD).

BCS	Phase	n	P_4_ (nmol/L)	p BCS	p Phase
BCS 2 (2-2.99)	Follicular	10	0.45±0.92		
	Luteal	38	32.91±0.98		
	Total	48	26.99±15.04		
BCS 3 (3-3.99)	Follicular	10	0.45±0.45		
	Luteal	38	30.97±1.11		
	Total	48	24.46±15.14	0.92	
BCS 4 (4-5.00)	Follicular	10	0.45±2.13		
	Luteal	38	31.42±1.11		
	Total	48	25.76±1.53		
Phase	Follicular	35	1.65±0.92**		0.00**
	Luteal	125	31.77±0.96**		
	Total	160	25.79±15.07		

** Total means with different superscripts within a row differs significantly (p<0.01). BCS=Body condition score, P_4_=Progesterone

These data were obtained during one whole estrous cycle. All steroid hormones were present at varying and significantly different (p<0.05) levels in each phase of the estrous cycle. Concentrations of progesterone and cortisol were higher (p<0.05) in the luteal phase, with progesterone level at the follicular phase was 1.65±0.92 nmol/L and 31.77±0.96 nmol/L in the luteal phase and cortisol level at the follicular phase was 70.95±17.68 nmol/L and 80.99±32.79 nmol/L at the luteal phase, as shown in Tables-5 and 7. Whereas estrogen levels were significantly higher in the follicular phase, as shown in Table-4, with estrogen level at the follicular phase was 339.12±72.22 pmol/L and 241.13±44.72 pmol/L at the luteal phase.

## Discussion

### Effect of BCSs on blood biochemical profiles

Body energy reserves, mainly represented by body fat and muscle content, are important determinants of reproductive performance [11]. A previous study showed that goats with a positive energy balance and a tendency toward emaciation were associated with better reproductive performance than goats with a normal metabolic state (BCS of 3-3.5); however, the endocrine mechanism underlying such associations requires elucidation [12]. These findings are similar to those observed in the present study. The results show that the BCS could affect blood protein concentration in Saanen goats a finding that was similar to that of previous studies [12-17], with higher protein levels in groups with a higher BCS (Table-1). In the present study, the blood glucose concentrations (Table-2) were also affected by the BCS factors [4]. The glucose level in group BCS 3 was higher than that in the other groups. Animals with higher glucose levels had more energy so that could be used for reproductive performance efficiently, with a BCS of 3 being better than that of groups with lower and higher BCSs, as also shown by the previous studies [3,4]. In this study, serum BUN concentrations (Table-3) were also significantly affected by the different BCS groups [18]. In BCS 2 animals, urea level was higher than that in animals with a higher BCS. This finding might be attributed to the fact that in group BCS 2, endogenous N compounds were catabolized to cope with an energy shortage. In this group, adaptive mechanisms to save as much N as possible were observed and contributed to the recycling of urea to the rumen [18,19]. Serum cholesterol concentrations (Table-6) were not significantly different among BCS groups and between the phases of the estrous cycle, similar to the finding of a previous study [4]. Cholesterol levels in dairy goats are approximately 3.33-7.22 mmol/L [20,21], which is similar to the findings of this study. Furthermore, there were no significant differences in the blood metabolites in each phase of the estrous cycle as also shown by the previous studies [22-25].

### Effect of BCSs on steroid hormone levels

The present study showed no significant differences in the peripheral serum progesterone levels in the three BCS groups (Table-7), as also shown in a previous experiment with cows [12]. A slightly lower progesterone concentration was observed in goats with a higher BCS, similar to the results of a previous study, in which the concentration of circulating progesterone was reduced in sheep with a positive energy balance [26]. Serum progesterone concentration was significantly higher (p<0.01) (Table-7) in the luteal phase than in the follicular phase. In cyclic goats, progesterone levels reduced to reach a minimum concentration during the follicular phase and then gradually increased to reach the maximum concentration in the luteal phase [27-29]. The present study found that progesterone concentrations were 1.65±0.92 nmol/L in the follicular phase and 31.77±0.96 nmol/L in the luteal phase, which was similar to those reported in a previous study [30]. The high progesterone levels observed in this study, which were above 15.90 nmol/L, might have resulted from the goats having more than one corpus luteum [31]. The estrogen level (Table-4) in group BCS 3 was the highest, with an average of 277.52±71.55 pmol/L. This group also had the highest values (355.72±68.98 pmol/L), compared to the others, at the follicular phase. Therefore, it could be suggested that the performance of BCS 3 goats was better than that of goats in the other BCSs groups [12,32]. The BCS of the goats showed a weak but direct positive correlation with the leptin levels [33]. Furthermore, lower estrogen production was associated with increased leptin concentration, which suppresses steroidogenesis in obese goats [32]. However, Sirotkin *et al*. [12] reported that leptin inhibited estradiol production in BCS 2 cattle and promoted estradiol release in BCS 3 cattle’s granulosa cells. Positive energy balance leads to increased fertility, with increased IGF-I release stimulating and enhancing steroidogenesis and folliculogenesis in the ovaries [12], and increasing the estrogen level. Hormone receptors could be affected by BCS [12]. Signaling molecules such as cyclic nucleotides, protein kinase [12,34], and transcription factors play an important role in regulating ovarian function and mediating the activity of various hormones. The serum estrogen level was significantly higher (p<0.01) (Table-4) in the follicular phase than in the luteal phase. The increase in estrogen level is associated with follicular development [35]. The peak values of estrogen generally correspond to the estrous cycle phases, and the present study found that the estrogen concentration in the follicular phase and luteal phase was 339.12±72.22 pmol/L and 241.13±44.72 pmol/L, respectively, similar to the concentration reported in a previous study by Damascus goats [28].

Cortisol profile in ideal (BCS 3) or poor BCS (BCS < 3; BCS > 4) remains unknown [36]. Cortisol stimulates the appetite, which is necessary to compensate for low body weight. Increased cortisol levels result in increased mobilization of energy, inhibition of protein synthesis, and reduction in body mass due to decreased structural tissue maintenance to fulfill energy requirements [11]. Elephants with lower BCSs showed a higher potential to increase stress hormone levels [37], which also occurred in obese animals. Similar results were obtained in the present study, where the cortisol level in group BCS 3 was the lowest among all groups, including animals with a higher or lower BCS (p>0.01), similar to the results in elephants [37]. Cortisol stimulates activation of the hypothalamic-pituitary-adrenal axis, which is associated with the inhibition of gonadotropin secretion and disruption of ovarian function [38]. An acute increase in cortisol levels suppresses luteinizing hormone pulse frequency [39]. In the present study, the serum cortisol level was higher (p<0.05) (Table-5) in the luteal phase than in the follicular phase, similar to the findings of a previous study [40]. Cortisol plays a role in preventing excessive uterine PGF_2a_ production and protecting the corpus luteum against apoptosis in non-pregnant ruminants [41]. The present study provides, for the 1^st^ time to our knowledge, data on blood metabolic and hormonal profiles in Saanen goats in Indonesia. Moreover, this study could improve the knowledge about the physiological parameters of this breed and motivate further research. The data collected in this study could be used to monitor and evaluate physiological status of the animals at the farmer level and could also improve the management of the breed.

## Conclusion

Concentrations of the metabolite and steroid hormones in goats such as protein, glucose, BUN, estrogen, and cortisol were affected with the BCS, and goats with BCS 3 had the ideal concentration of those variables rather than that with the lower and the higher BCS. Furthermore, the concentration of steroid hormones was affected by the estrus phase. However, in this study, no significant differences were observed in the metabolite blood profile levels due to the phase of estrous cycle.

## Authors’ Contributions

PIS and DTW conceived, conducted the fieldwork, administrated, and drafted the manuscript. PIS conducted the literature search. All authors conducted data interpretation and edited the manuscript. DTW, BPW, and SB designed and supervised the study. PIS and DTW performed the statistical analysis and reviewed the manuscript. All authors read and approved the final manuscript.
